# Anxiety, insomnia, and depression during COVID-19 lockdown in elite individual and team sport athletes

**DOI:** 10.1186/s41155-022-00233-z

**Published:** 2022-10-10

**Authors:** Jose I. Salles, Carolina Silva, Aline Wolff, Ludmilla Orwert, Pedro Ribeiro, Bruna Velasques, Dylan Morrissey

**Affiliations:** 1 Sports and Exercise Medicine, William Harvey Research Institute, QMUL, Bancroft Road, London, E1 4DG UK; 2 Olympic Laboratory, Brazilian Olympic Committee, Av. Embaixador Abelardo Bueno, 851 Barra da Tijuca, Rio de Janeiro, CEP 22775-040 Brazil; 3 NHS Trust, Surrey, UK; 4 Brain Mapping and Sensorimotor Integration Laboratory, Institute of Psychiatry of the Federal University of Rio de Janeiro, Av. Venceslau Brás, 71 - Botafogo, Rio de Janeiro, CEP 22290-140 Brazil; 5 Neurophysiology and Neuropsychology of Attention, Institute of Psychiatry of the Federal University of Rio de Janeiro, Av. Venceslau Brás, 71 - Botafogo, Rio de Janeiro, CEP 22290-140 Brazil

**Keywords:** COVID-19, Anxiety, Depression, Insomnia, Team sports, Individual athletes

## Abstract

**Purpose:**

Pandemic-induced lockdowns disrupted sport training and competition. We aimed to identify the impact on the mental health of high-level athletes and clarify whether the effects differ for team-based and individual athletes.

**Methods:**

This cross-sectional survey, stratified by sex and sport type, collected demographic data and mental health measurements from 274 Brazilian high-performance athletes (142 from team sports and 132 from individual sports) involved with the Brazilian Olympic Committee program for the Tokyo Olympics 2021. Depression, disturbed sleep, and anxiety were assessed by the 9-Item Patient Health Questionnaire, 7-Item Insomnia Severity Index, and 7-Item Generalized Anxiety Disorder scale respectively. Responses were analyzed dichotomously according to published threshold values, characterizing the relative frequency distribution of prevalence (PCRS) or non-prevalence of clinically relevant symptoms (NPCRS).

**Results:**

Out of all participants, 47 [17.1%], *Z*(274) = 15.38, *p* = .001, 32 [11.7%], *Z*(274) = 17.94, *p* = .001, and 49 [17.9%], *Z*(274) = 15.04, *p* = .001 had PCRS of anxiety, insomnia, and depression, respectively. There were no significant differences in the PCRS among genders. Compared with individual sport athletes, team sport athletes were more likely to report PCRS of insomnia (12 [37.5%] vs 20 [62.5%], *Z*(274) = −2.00, *p* = .046), and depression (18 [36.7%] vs 31 [63.3%], *Z*(274) = −2.63, *p* = .009) but not for anxiety.

**Conclusion:**

Athletes reported high levels of mental health problems during the lockdown. Team sport athletes reported worse symptoms of insomnia and depression than individual sport athletes, possibly due to the impact of unaccustomed social isolation and lack of social team activity. Therefore, it becomes relevant to consider psychological support to team sport athletes who for some reason, such as a pandemic, enduring crisis even injury rehabilitation needs to be isolated.

## Introduction

The coronavirus (COVID-19) outbreak in 2019 rapidly became a public health emergency of international concern impacting multiple aspects of everyday life across the world (The Lancet, 2020; WHO, 2020). During the tropical winter of 2020, the coronavirus disease affected a significant proportion of the population across a wide geographic area in Brazil, and like most countries, worldwide Brazilian citizens were subjected to restrictions of free movement and instructed to stay at home to ensure the effective reduction of new cases, transmissions, and deaths (Mayr et al., 2020). Research and case studies have shown that lockdowns are effective at reducing the spread of COVID-19 (Perra, 2021). Given the concerns about the evolutionary nature of the pandemic and the increasing spread of COVID-19, all sport competitions, including the Tokyo 2020 Olympic and Paralympic Games were postponed and the training of athletes markedly disrupted. The associated restrictions on the quality and quantity of training and competition resulted in the loss of hard-won physical fitness and deterioration in performance ability (Jukic et al., 2020; Ambroży et al., 2021). These effects on sporting ability, in combination with the adverse events on individuals in society, may have led to frustration, anger, anxiety, and depression even in the most resilient athletes (Pons et al., 2020; Toresdahl & Asif, 2020; Leguizamo et al., 2021; Gupta & McCarthy, 2021; Pellino et al., 2022).

The increasing interest in elite athlete mental health is reflected in recent consensus statements outlining mental health symptom identification and management in this population (Gouttebarge et al., 2021). The highest-level athlete environment produces psychologically unique challenges and numerous sacrifices due to the nature of the sport career such as contracts with clubs and sponsors, economic volatility, musculoskeletal injuries, fear of failure in competition, and the constant mental effort required to succeed at the international level (Grupe & Nitschke, 2013; Nixdorf et al., 2016; Rice et al., 2016). Although some studies have highlighted depression and anxiety among elite athletes (Castro-Sánchez et al., 2018; Elbe & Jensen, 2016; Nixdorf et al., 2013; Wolanin et al., 2016), it is expected that the global lockdown has resulted in loneliness and triggered mental health issues; however, it may also have been the case that the suspension of training and competition may have given some time for rest and recovery.

According to the International Olympic Committee consensus statement, mental health symptoms and disorders are common in elite athletes (Reardon et al., 2019). Athlete-specific factors may cause or exacerbate anxiety and depression disorders (Du Preez et al., 2017; Gorczynski et al., 2017; Gouttebarge et al., 2019; Rice et al., 2019). Previous studies have reported the prevalence of generalized anxiety disorder of 6% among French elite athletes (Schaal et al., 2011) and 15% prevalence of depression among German high-level athletes (Nixdorf et al., 2013). During the COVID-19 pandemic lockdown, depressive symptoms and insomnia were present in 6% and 4%, respectively, of a Swiss elite athletes’ cohort (Fröhlich et al., 2021).

Mental health differs according to the type of sports in which an athlete participates. Previous research has identified that elite athletes participating in an individual sport are at a higher risk for depressive symptoms than team sport athletes (Nixdorf et al., 2013, 2016; Elbe & Jensen, 2016; Wolanin et al., 2016). Under lockdown conditions, team athletes endured training modifications that can lead to contributing to anxiety and depression (Chen et al., 2020; Schinke et al., 2020) as their performance improvement is teammate-related in training while individual sport athletes are used to training independently. Although there exist anxiety, depression, and insomnia disorder studies in athletes’ populations (Chen et al., 2020; Schinke et al., 2020), to our knowledge, there has been no research on Brazilian elite athletes in this field. Therefore, our general objective was to describe the degree of clinically relevant anxiety, insomnia, and depression symptoms in a high-performance cohort of Brazilian athletes during the COVID-19 lockdown with a particular focus on differences between individual and team ball sports. We hypothesized that (i) high levels of anxiety would be accompanied by depression and insomnia symptoms and (ii) athletes from team ball sports would be particularly affected compared to individual athletes.

## Methodology

### Participants

Seven hundred thirty-two elite Brazilian athletes involved in the Brazilian Olympic Committee training program targeting the 2020 Tokyo Olympics were invited to participate in a survey designed to investigate depression, anxiety, and insomnia during the first COVID-19 lockdown in Brazil. Of these, 274 athletes agreed to participate, of which 41% effectively attended the Games in the 2021 Olympics. They were from individual sports: athletics (*n* = 74), gymnastics (*n* = 8), swimming (*n* = 50), and from team ball sports: basketball (*n* = 11), football (*n* = 5), handball (*n* = 52), rugby (*n* = 22), volleyball (*n* = 30), and water polo (*n* = 22). The mental health survey study was via email with athletes being routed to an online survey available for 4 weeks in August 2020. At the time, Brazilian nationwide contact restrictions at the time were still being implemented by decree where people were urged to reduce social contacts to a minimum (Decreto No. 65,114, 2020). The online survey research used questionnaires including demographic questions, in addition to 3 valid and reliable mental health research instruments. This study protocol was approved by the Federal University of Rio de Janeiro Institutional Ethics Committee Board and all subjects provided informed consent electronically (CEP 32628820.3.0000.0008).

### Instruments and procedures

#### Socio-demographic measures

Socio-demographic information was collected including the participant’s age, gender, type of sport participation was classified as either an individual sport or a team sport, and weekly training duration per week before and during the lockdown.

#### Mental health assessment questionnaire

##### Insomnia Severity Index (ISI)

The ISI is a 7-item self-report questionnaire assessing the nature, severity, and impact of insomnia over the last 2 weeks in initiating and maintaining sleep, waking up early, interference with routine activities, and satisfaction with sleep patterns (Morin et al., 2011). A 5-point Likert scale is used to rate each item assigned according to the response and the sum of the points classifies the occurrence and severity of insomnia. The total score is interpreted as follows: an absence of insomnia (0–7), sub-threshold insomnia (8–14), moderate insomnia (15–21), and severe insomnia (22–28). For the present study, we opted to dichotomize the scores. All athletes with 15 or more points on the ISI were considered to have a prevalence of clinically relevant symptoms of insomnia (Castro et al., 2009). We applied psychometric properties for the Brazilian Portuguese version of ISI (Castro et al., 2009) administered in the recent study carried out in Brazil (Brito-Marques et al., 2021). High internal consistency coefficients were obtained from a community sample for an epidemiological study of insomnia (Cronbach *α* = 0.91) (Morin et al., 2011).

##### The General Anxiety Disorder 7-Item (GAD-7)

The scores on the GAD-7 scale are determined through participant responses to a 7-item anxiety questionnaire using a 4-item Likert rating scale ranging from 0 (not at all) to 3 (almost every day), such that the total score ranges from 0 to 21 (Toussainta et al., 2020). The GAD-7 was used to assess how often participants had been bothered by seven potential problems over the past 2 weeks. The test result classifies the individual as having the following levels of anxiety: normal (0–4), mild (5–9), moderate (10–14), and severe (15–21) anxiety symptoms. The cut-off score ≥10 was considered as screening for moderate to severe anxiety and was used to determine the existence of prevalence of clinically relevant symptoms of anxiety among participants (Bergerot et al., 2014). The GAD-7 was validated by Kroenke et al. (2007), and the translation into Portuguese was carried out by Pfizer (Copyright © 2005 Pfizer Inc., New York, NY), with evidence of validity registered in Brazil (Bergerot et al., 2014). Dhira et al. (2021) showed excellent reliability of GAD-7 as measured by Cronbach’s *α* (0.895).

##### 9-item Patient Health Questionnaire (PHQ-9)

This is a 9-item screening questionnaire from the complete Patient Health Questionnaire for depression symptoms (Kroenke et al., 2001). Participants were requested to report symptoms experienced during the 2 weeks before completion, with the questionnaire being scored using a 4-point severity scale (0 = not at all, 1 = several days, 2 = more than half the days, 3 = nearly every day). Therefore, the total scores range between 0 and 27 (0–4 = minimal or none, 5–9 = mild, 10–14 = moderate, 15–19 = moderately severe, 20–27 = severe), and in the current study, a cut-off score ≥10 was considered to indicate the prevalence of clinically relevant symptoms among participants (Osório et al., 2009). The PHQ-9 was translated into Portuguese by Pfizer (Copyright © 2005 Pfizer Inc., New York, NY) (Bergerot et al., 2014). Rahman et al. (2022) showed excellent reliability of PHQ-9 as measured by Cronbach’s *α* (0.824).

### Data and statistical analysis

Demographic data (age group, sex, type of sport, type of training during the lockdown) and the severity of psychological depressive disorders, disturbed sleep, and anxiety were calculated as absolute values and percentages. To compare the total number of weekly training hours before and during the lockdown in each type of sport (individual and team ball sports) and the number of athletes by gender in each type of sport, the Wilcoxon test was performed with an *α* level of 0.05.

A Kolmogorov–Smirnov test was conducted, as we found that the original scores of the 3 questionnaires were not normally distributed and therefore were presented as mean, median, and interquartile ranges. The correlation between the three questionnaires was assessed with the Spearman rank test.

The GDA-7, ISI, and PHQ-9 questionnaires were analyzed dichotomously according to their cutoff values, characterizing the relative frequency distribution (percentages) of prevalence (PCRS) or non-prevalence of clinically relevant symptoms (NPCRS) throughout the sample, where the test of equality of proportions was applied including the specific assessment of the athletes with PCRS according to gender and type of sport. *P* values < 0.05 indicated that a difference was statistically significant. The data were analyzed using SPSS (IBM SPSS Statistics for Windows, Version 20.0. Armonk, NY: IBM Corp).

## Results

The 274 participants completed the survey by providing informed consent electronically. The athletes tended to be male (59.9%), with a mean age of 25.6±4.9 with 64% aged 18 to 27 years. There was only a slight percentage tendency in the number of athletes toward the team ball sports (3.6%), indicating the non-prevalence of a type of sport, individual or team ball sports. There was a tendency for athletes to choose physical training (33.9%) compared to physical training combined with technical training (23.4%) during the lockdown (Table 1). The athletes reported more training hours per week spent before (22.2 ± 5.2) than during lockdown (11.0 ± 4.1) *Z*(274) = −14.39; *p* =.001. The individual athletes reported more training hours per week spent before (23.5 ± 5.8) than during lockdown (13.3 ± 4.1), Z(274) = −9.99; *p* =.001. The same was observed with team ball sport athletes who reported to have undergone more training hours per week before (21.0 ± 4.2) than during lockdown (8.9± 2.8), *Z*(274) =−10.37; *p* =.001. There were no significant differences in the proportions of female and male athletes’ number among the type of sports (Table 1), *X*^2^ (2, *N* = 274) = 0.55, *p* =.458.

**Table 1:** Demographic data of individual and team sport athletes

Descriptive variables	Sub-variables	*N*	%
Age range (*n* = 274, mean age 25.6±4.9)	18 to 22	83	30.3
	23 to 27	93	33.9
	28 to 32	66	24.1
	33 to 37	28	10.2
	38 to 42	4	1.5
Sex	Female	110	40.1
	Male	164	59.9
Type of sport played	Individual sports	132	48.2
	Team sports	142	51.8
Type of sports per sex	Female individual sports	56	42.4
	Female team sports	54	38.0
	Male individual sports	76	57.6
	Male team sports	88	62.0
Training focus during lockdown	Physical training	93	33.9
	Technical and physical training	64	23.4
	Technical, physical, and mental training	48	17.5
	Physical and mental training	44	16.1
	Technical training	20	7.3
	Technical and mental training	4	1.5
	Mental training	1	0.4

Individual sports: athletics, gymnastics, and swimming; team sports: basketball, football, handball, rugby, volleyball, and water polo

**Fig. 1:**
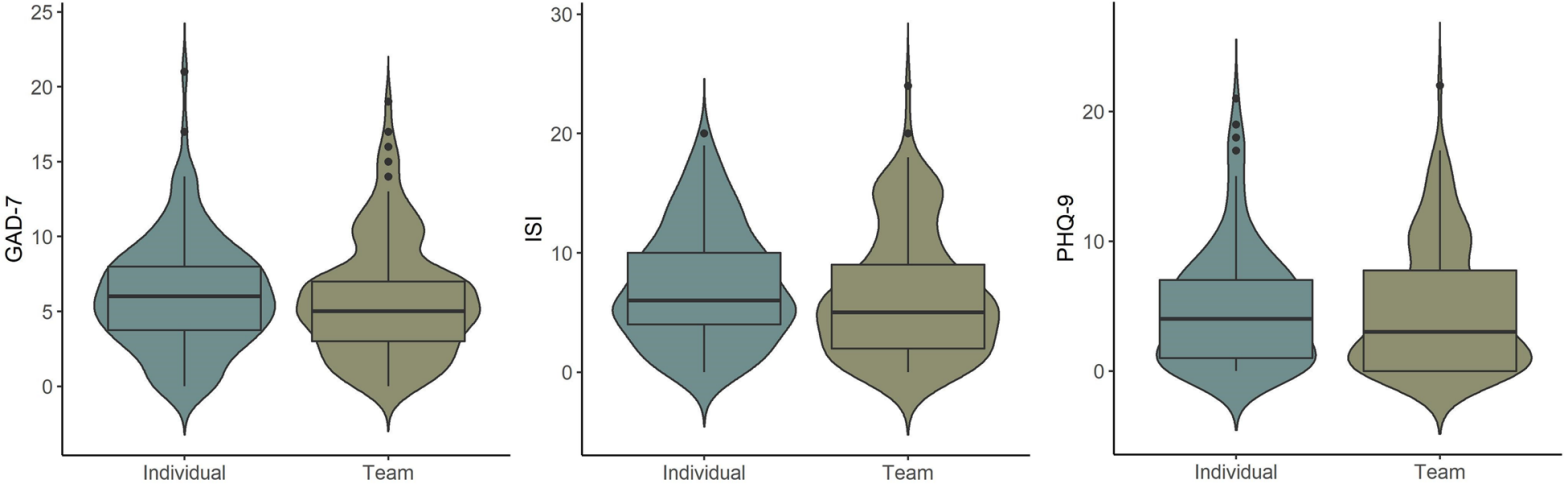
Individual and team ball sport mental health questionnaires scores. Legend: GAD-7, 7-Item Generalized Anxiety Disorder; ISI, 7-Item Insomnia Severity Index; PHQ-9, 9-Item Patient Health Questionnaire

The mean, median, and interquartile scores on the GAD-7 for anxiety (5.97, 5 [3-8]), ISI for insomnia (6.85, 6 [3-10]), and PHQ-9 for depression (4.80, 4 [1-7]), for all respondents, are shown in Fig. 1.

**Fig. 2:**
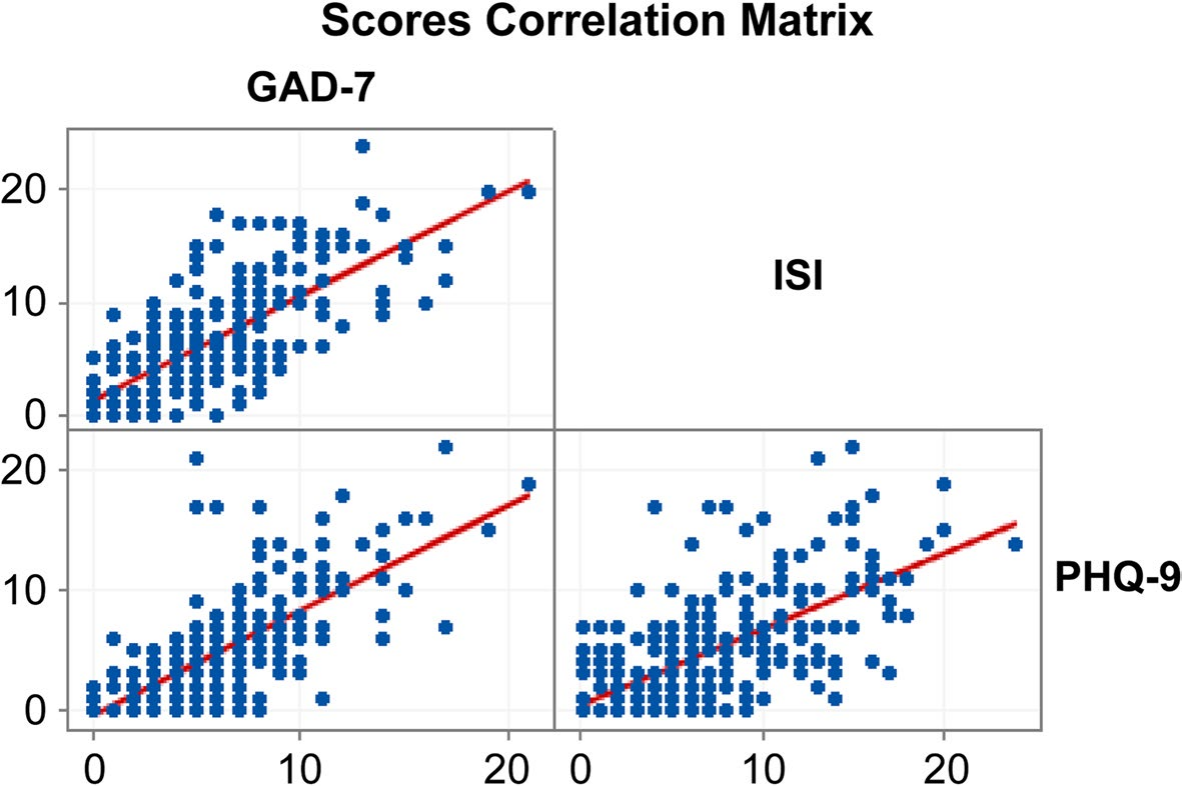
Mental health questionnaire correlation. Legend: GAD-7, 7-Item Generalized Anxiety Disorder; ISI, 7-Item Insomnia Severity Index; PHQ-9, 9-Item Patient Health Questionnaire

The three questionnaires applied in the present study have a statistically significant correlation between their scores. The correlations were positive, where GAD-7 and PHQ-9 have a high correlation *r*(274) = .707; *p* =.001, both PH-9 and ISI *r*(274) = .674, *p* =.001 and GAD-7 and ISI *r*(274) = .692, *p* =.001 have moderate correlation (Mukaka, 2012) (Fig. 2).

**Fig. 3:**
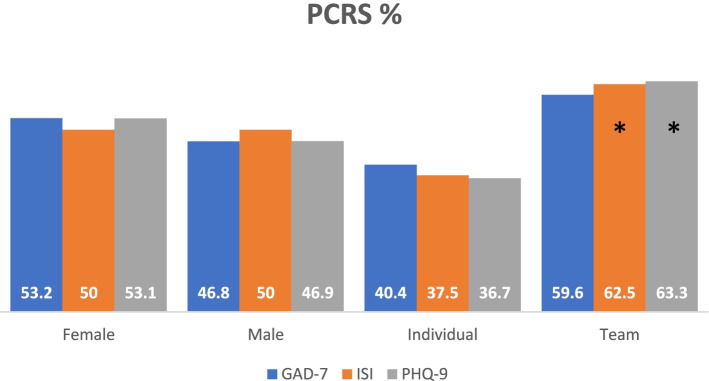
Athlete distribution with PCRS per questionnaire comparing sex and types of sports played. Legend: GAD-7, 7-Item Generalized Anxiety Disorder; ISI, 7-Item Insomnia Severity Index; PHQ-9, 9-Item Patient Health Questionnaire

A low percentage of athletes had prevalence of clinically relevant symptoms of anxiety: 47 [17.1%] *Z*(274) = 15.38, *p* =.001, insomnia 32 [11.7%], *Z*(274) = 17.94, *p* =.001, depression 49 [17.9%], *Z*(274) = 15.04, *p* = .001, during the lockdown (Table 2). There were no significant differences in the GAD-7, ISI and PHQ-9 scores among the sex of athletes with the prevalence of clinically relevant symptoms, as shown in Fig. 3. Compared with individual sport athletes, team ball sport athletes were more likely to report the prevalence of clinically relevant symptoms of insomnia 12 [37.5%] vs 20 [62.5%], *Z*(274) = −2.00, *p* =.046, and depression 18 [36.7%] vs 31 [63.3%], *Z*(274) = −2.63, *p* =.009 but not for anxiety 19 [40.4%] vs 28 [59.6%], *Z*(274) =−1.86, *p* =.063 as shown in Fig. 3.

**Table 2:** Prevalence or non-prevalence of clinically relevant symptoms

Questionnaires	ISI		GAD-7		PHQ-9	
	***N***	%	***N***	%	***N***	%
**NPCRS**	242	88.3	227	82.8	225	82.1
**PCRS**	32	11.7*	47	11.7*	49	17.9*

*NPCRS* Non-prevalence of clinically relevant symptoms, *PCRS* prevalence of clinically relevant symptoms, *ISI* 7-Item Insomnia Severity Index, *GAD-7* 7-Item Generalized Anxiety Disorder, *PHQ-9* 9-Item Patient Health Questionnaire

*p = .001

## Discussion

The symptoms of depression, anxiety, and sleep disturbance among athletes previously described in the literature (Gupta et al., 2017; Kilic et al., 2021; Nixdorf et al., 2016; Schaal et al., 2011) are here associated with COVID-19-related descriptions of worry in the sport context. Anxiety, insomnia, and depression may be caused by worry about becoming infected and lifestyle changes (Zhang et al., 2020). Findings from the present study suggest a prevalence rate in elite athletes for anxiety, insomnia, and depression symptomatology of 17.2%, 11.7%, and 17.9%, respectively. Considering that the global population is affected with a higher prevalence rate of depressive and anxiety symptoms of about 4.5% (WHO, 2017) and 6% (Wittchen & Jacobi, 2005), respectively, we can no longer assume that psychosocial benefits attributed to the sports may serve to protect against the development of psychological disorders (Elbe & Jensen, 2016; Gouttebarge et al., 2021). We further ascertained that a significant correlation took a place between anxiety, insomnia, and depressive symptoms among athletes. Therefore, as hypothesized, the study demonstrated that athletes with high levels of anxiety experienced more depressive and disturbed sleep symptoms than athletes with lower levels of anxiety during the lockdown.

### Mental health and type of sport participation

In usual circumstances, athletes in individual sports are more likely to be diagnosed with a psychological problem than athletes in team ball sports. This is based on the premise that individual sport athletes require more internal attributions as they do not have teammates to share positive or negative sport performance and the failure becomes non-transferable. A team ball sport athlete is, therefore, less likely to take responsibility for an unsuccessful action compared to individual sport athletes. These circumstances are proposed to explain the higher levels of anxiety and depression in individual athletes compared to team sport athletes (Nixdorf et al., 2016; Schaal et al., 2011). However, in the present study, the elite athletes may have experienced disappointment and irritability under the social isolation measures during the COVID-19 pandemic leading to a higher percentage of team ball sport athletes reporting high values of depression and insomnia albeit they did not differ significantly in terms of anxiety.

One reason for such a high prevalence of depression may be the perception of loneliness and feelings of guilt over unfortunate factors during negative coping strategies of resignation that variably affect the development of depression (Brooks et al., 2020). This may be exacerbated for team ball sport athletes due to the absence of social interactions with teammates, including the core services commonly delivered from the staff team for their players. Furthermore, the presence of residual poor sleep among athletes from team ball sports being associated with depressive symptoms (Asarnow & Manber, 2019) was also identified in the present study. Accumulating evidence has shown that during depressive episodes adults report difficulties initiating or maintaining sleep (Ford & Kamerow, 1989; Sadler et al., 2018). On the other hand, anxiety is a common emotional state experienced by athletes when they are facing worrying thoughts and apprehensions (Gu et al., 2020) and during the COVID-19 outbreak was likely associated with the uncertainties in many future aspects of athlete's professional lives both team and individual sports.

Our findings suggest that individual sport athletes are used to being able to train independently of teammates and coaches and a sudden shift for the team sport athletes to solo exercise was frustrating. Previous psychological research comparing team and individual athletes demonstrated that team sports demand greater competitiveness as well as cooperation than individual sports concluding that cooperative behavior can be an extremely important difference between both types of sport athletes (Landkammer et al., 2019). The team sport athletes are conditioned to improve their perceptions to be able to cooperate meaningful information to coordinate technical and tactical actions with their teammates (Steiner et al., 2017). In other words, it is assumed that significant athletic environment variations according to the type of sports practiced combined with a particular personality and genetic predisposition may trigger stress response leading to psychological health.

### Sex and mental health

According to our data, there was no difference between male and female athletes among those that reported a prevalence of clinically relevant symptoms levels of anxiety, insomnia, and depression during the lockdown. Previous research has reported higher rates of anxiety, sleep problems, and depression in female athletes than in males (McGuine et al., 2020; Schaal et al., 2011; Wolanin et al., 2016). Our contradictory findings indicate that female athletes may have found coping strategies that offset the extra pressures of the pandemic, such as maintaining social networks and being inclined to talk about their emotional symptoms (Schaal et al., 2011). Substantial evidence suggests that social support is an important external resource to safeguard against the negative effects of stressors (DeLongis & Holtzman, 2005), and among women, it is known to reduce the risk of major depression compared to men (Kendler et al., 2005). Therefore, our results support the concept that the pandemic restraints, when associated with gender, might play a role in the development of mental disorders in male athletes.

### Limitations

Our research also has some limitations. First, the team sport participants group did not include all team sports, potentially limiting the generalization of our findings to team ball sports. Second, compared with face-to-face interviews, measurement of mental health through self-report in online surveys might have limitations compared to the traditional oral clinical examinations conducted by mental health professionals. Third, we were unable to distinguish pre-existing to comped to new mental health symptoms. Finally, the cross-sectional design meant the efficacy of sport psychology services during the pandemic period was not assessed. Finally, no research on mental health in Brazilian athletes has been applied which limits us to make inferences from our data. In addition, through our sample, we were unable to distinguish pre-existing mental health conditions compared to the prevalence of clinically relevant symptoms of anxiety, depression, and insomnia during the confinement period. It leads us to suggest future studies assess high-performance Brazilian athletes’ mental health without social restrictions.

## Conclusion

In this survey study of elite athletes who responded about how they coped with the lockdown period, a significantly higher proportion of athletes who presented with clinically relevant symptoms of a mental disorder were from teams compared to individual sports. An absence of organized training and competition and a lack of adequate communication between teammates or their coaches may explain why team sport athletes were found to be more susceptible to clinically relevant mental health symptoms compared to individual sport athletes. It reinforces our hypothesis and leads us to consider as a practical implication that support from the team staff and teammates may be a factor that restrains psychopathologies and needs to be enhanced during lockdown periods and social isolations due to recovery from injury or illness. It is therefore particularly important to examine which sources and mechanisms may contribute to such mental health disorders. In further investigations, the research could investigate such causal mechanisms in prospective study designs where the teaching of individual coping strategies, and organizational support structures, may need to be implemented for team sport athletes.

## Data Availability

The datasets generated during and/or analyzed during the current study are available from the corresponding author on reasonable request.

## Abbreviations

PCRS: Prevalence of clinically relevant symptoms
NPCRS: Non-prevalence of clinically relevant symptoms
COVID-19: Contagious respiratory disease caused by the SARS-CoV-2 virus
ISI: Insomnia Severity Index
GAD-7: General Anxiety Disorder 7-Item
PHQ-9: 9-Item Patient Health Questionnaire
ES: Effect size
